# Development of crizotinib-associated renal cyst in a non-small cell lung cancer patient with ALK fusion: a case report and review of the literature

**DOI:** 10.1186/s13000-024-01480-7

**Published:** 2024-04-14

**Authors:** Peng Zhang, JiaHua Xu, Qing Wu, Jianxin Qian, Song Wang

**Affiliations:** 1 Six Departments of Oncology, Longhua Hospital Affiliated to Shanghai University of Traditional Chinese Medicine, Shanghai, China; 2 Seven Departments of Oncology, Longhua Hospital Affiliated to Shanghai University of Traditional Chinese Medicine, Shanghai, China; 3 Department of Radiology, Longhua Hospital Affiliated to Shanghai University of Traditional Chinese Medicine, Shanghai, China

**Keywords:** Crizotinib, Non-small cell lung cancer, Crizotinib-associated renal cyst, Case report

## Abstract

**Background:**

Crizotinib, an oral first-generation tyrosine kinase inhibitor (TKI), is superior to systemic chemotherapy for the treatment of non-small cell lung cancer (NSCLC) with positive rearrangement of anaplastic lymphoma kinase (ALK). However, an increased incidence of renal and hepatic cysts has been reported in the patients on crizotinib treatment.

**Case presentation:**

Here, we describe a case of a 71-year-old Chinese women developed multiple cystic lesions in kidney and liver during crizotinib treatment for the primary and metastatic NSCLC. The renal and hepatic cysts were noted by CT scan 3 months after crizotinib treatment, which were spontaneously and significantly regressed after stopping crizotinib.

**Conclusions:**

Based on literature review and our experience in this case report, we concluded that crizotinib-associated renal cyst (CARCs) has features of malignancy and abscess in radiographic imaging, and thus, pathological confirmation is necessary to avoid inappropriate treatment decision. In addition, to benefit the patients with progress-free survival (PFS), switching from crizotinib to alectinib is recommended for the treatment of NSCLC patients who developed CARCs.

**Supplementary Information:**

The online version contains supplementary material available at 10.1186/s13000-024-01480-7.

## Background

Lung cancer is the predominant malignancy and the leading cause of cancer-related mortality with over 610,000 deaths in 2015 in China [1, 2]. Non-small cell lung cancer (NSCLC) accounts for approximately 85% of lung cancer [3, 4], and 3–5% of NSCLC has a gene rearrangement of *analplastic lymphoma kinase* (*ALK*) [5, 6]. Crizotinib, an oral first generation tyrosine kinase inhibitor of ALK, c-ros oncogene 1 (ROS-1), and c-met proto-oncogene product (c-MET) [7–10], significantly increased therapeutic response and progress-free survival (PFS) in patients with advanced NSCLC with *ALK* fusion or rearrangement in comparison with standard chemotherapy as first-line treatment [11, 12].

While the targeted anti-cancer therapy with tyrosine kinase inhibitors have resulted in dramatical improvement in patients’ survival compared to systemic chemotherapy, renal toxicities of targeted agents are increasingly being recognized. In this regard, the most common and known side effects of crizotinib are nausea, vision disorders, elevation of alanine and aspartate aminotransferase levels, and renal impairment [13]. While accumulating number of case reports on the crizotinib-associated renal cyst (CARC) haven been published since the first report by Lin et al. in 2014 [14], the etiology, natural history, and molecular mechanism of CARC are unknown.

Here, we report a case of multiple cystic lesions in kidney and liver in a patient who was treated with crizotinib after being non-responsive to systemic chemotherapy for the primary left central and metastatic NSCLC with positive *ALK* fusion. The size of the lung cancers was dramatically decreased after 2 months on crizotinib. However, multiple renal and hepatic cysts were noted after 3 months on crizotinib treatment, which continuously grew till discontinuation of crizotinib treatment at 5 months after initialization of crizotinib therapy. The cystic lesions were spontaneously and significantly regressed one month after stopping crizotinib.

## Case presentation

**Fig. 1:**
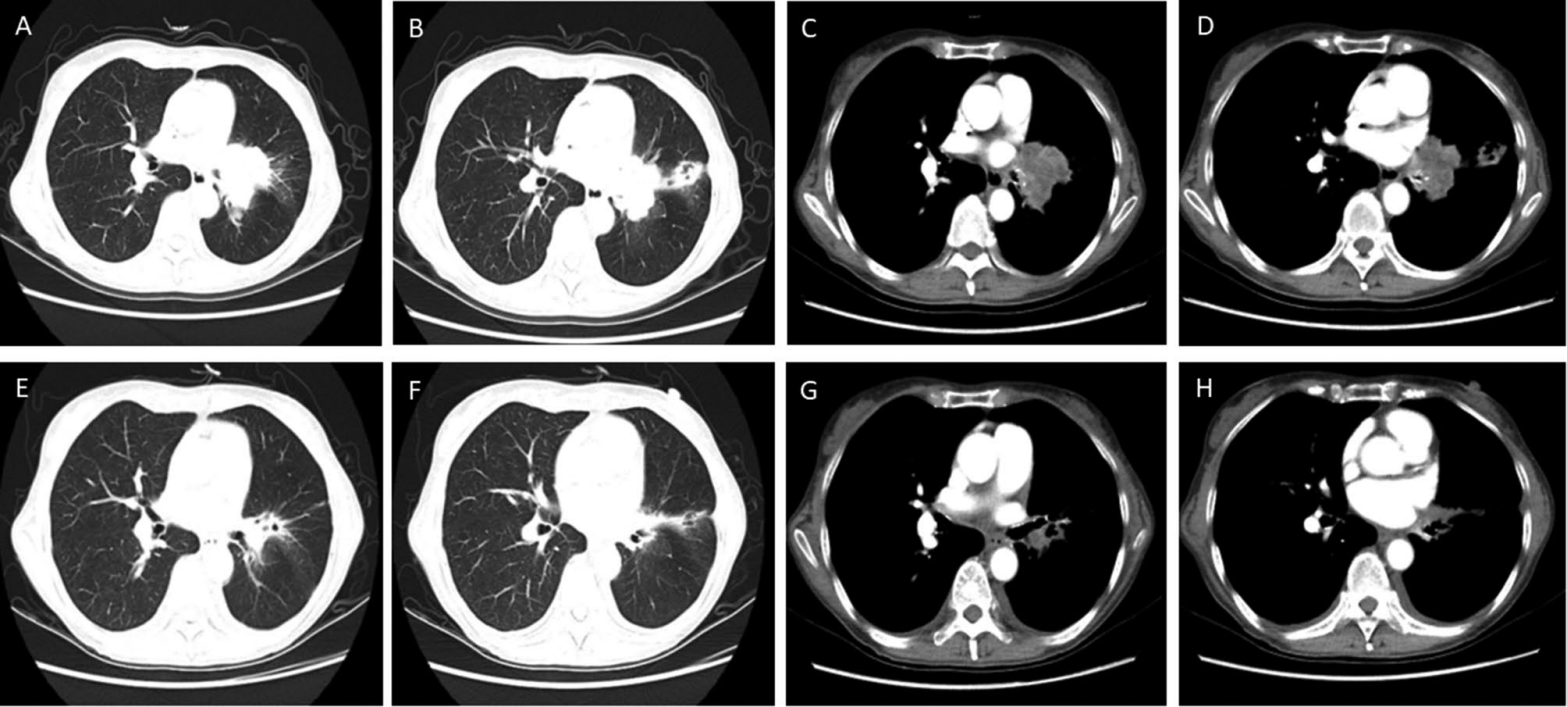
Contrast-enhanced chest CT scan before and after crizotinib treatment. **Panels A-D**: Prior to crizotinib treatment. **Panels E-H**: Post 2 months on crizotinib treatment, showing the size of primary tumor on left hilum significantly decreased

A 71-year-old Chinese woman was initially diagnosed as NSCLC through biopsy of a neoplastic tissue at left hilum on Dec 28th, 2015. Histology of the tumor revealed as adenocarcinoma with positive staining of cluster of differentiation 68 (CD68, +), carcinoembryonic antigen (CEA, +), thyroid transcription factor-1 (TTF-1, +), and Napsin A (+), but negative staining of P40 protein (-) and CD5/6 (-) by histochemistry. Genetic analysis indicated, epidermal growth factor receptor (EGFR, Exons 18, 19, 20, and 21): wild type; Kirsten rat sarcoma virus (KRAS, Condon 12 and 13): wild type; B-Raf proto-oncogene (BRAF, Exon 15): wild type; ALK: fusion type; and ROS Proto-Oncogene 1 (ROS1): wild type. Given the ALK fusion type, the patient was advised to treat with crizotinib. However, the patient and families declined the crizotinib regimen, and thus, a systemic chemotherapy (pemetrexed 700 mg intravenous injection on day 1, cisplatin 35.5 mg intravenous injection on day 1, 2, and 3) was initiated in December 2015 and completed the first full therapeutic course in April 2016. Chest CT of June 2017 indicated the malignancy increased in size, and thus, the second course of systemic chemotherapy was carried out from July through September 2017 with outcome of stabilizing disease. Nearly two years after completion of the systemic chemotherapy (May 2019), contrast-enhanced CT scan demonstrated metastasis of the central lung cancer to cervical spine, scrum, right ilium, and right side first anterior rib. Regimen of crizotinib (250 mg, bid po) plus periodic pamidronate disodium injection was then initiated immediately. After 2 months of the treatment with crizotinib, chest CT scan showed that the size of primary cancer on the hilum of left lung as well as the metastatic tumors were significantly reduced (Fig. 1). However, enlarged lymph nodes on the hilum of left lung (2.0 × 2.7 cm size), obstructive pneumonia of left lung, and mild pleural effusion on both sides were noticed.

**Fig. 2:**
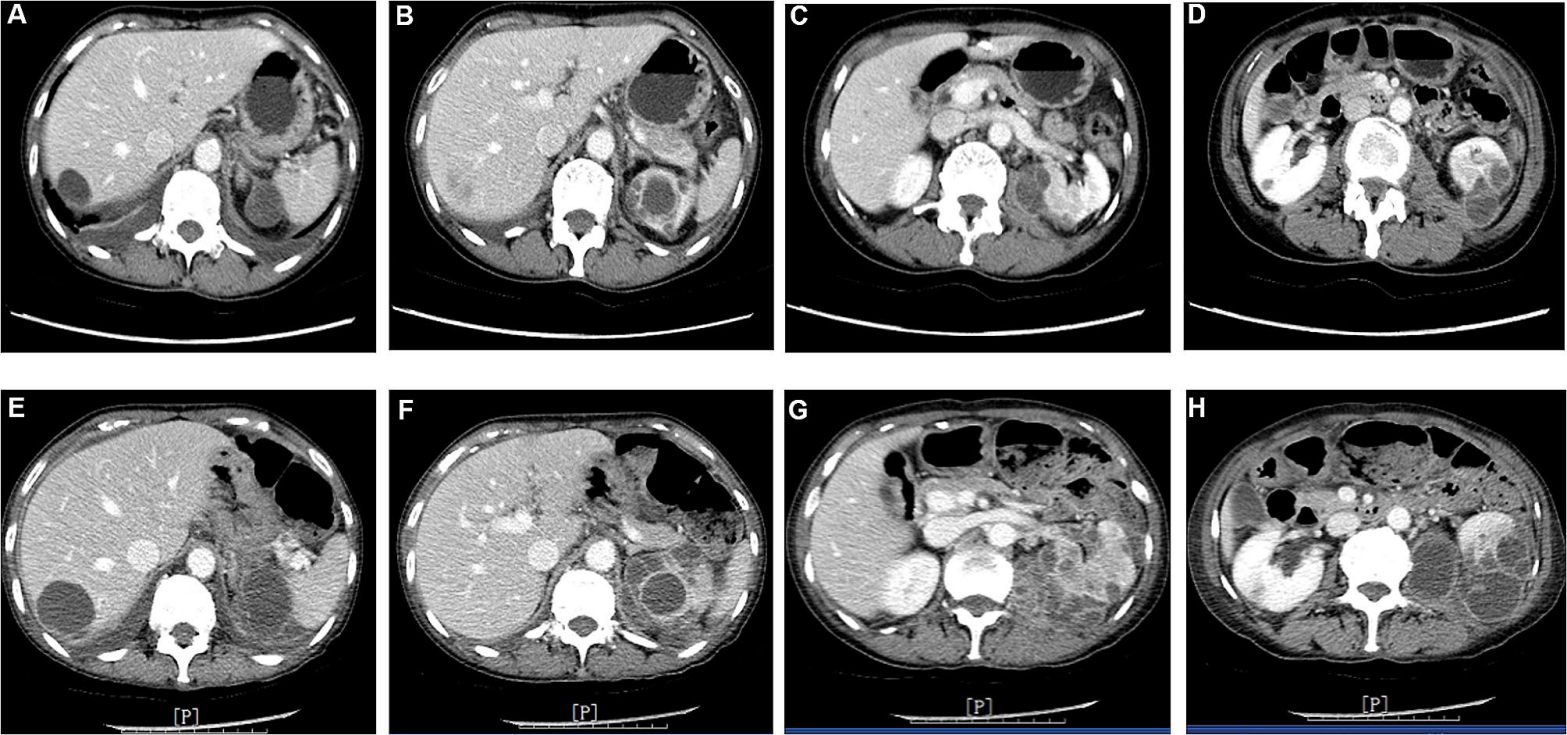
Contrast-enhanced abdominal CT scan during crizotinib treatment. **Panels A-D**: Post 3 months on crizotinib treatment, demonstrating the cystic lesions have been developed in the kidneys and liver. **Panels E-H**: Post 5 months on crizotinib treatment, demonstrating the cystic lesions in the kidneys and liver continuously grew during the additional 2 months of crizotinib treatment

After 3 months on crizotinib treatment, the patient was hospitalized in August 2019 due to low fever (38 °C), backpain, and fatigue. Laboratory examination on admission showed white blood cell count was 11.78 × 10^9^/L, and CT scan indicated multiple cystic masses on both kidneys and liver (Fig. 2A-D). Considering potentiality of metastatic tumors, needle biopsy for the kidney cystic mass was performed and approximately 10mL pus fluid was drained from the cystic mass during the procedure. Pathological examination of the biopsy tissues and drainage from the cystic mass on the left kidney revealed granulation tissues with mild infiltration of lymphocytes and negative malignancy. Immunohistochemistry of the biopsy tissue indicated TTF-1 (-), cytokeratin antibodies AE1/AE3 (-), Ki-67 protein (5%+), cytokeratin 7 (CK7) (-), Paired-box gene 8 (PAX-8, -), P40 protein (-), and Villin (a calcium-regulated actin-binding protein, -). To control fever, moxifloxacin and imipenem cilastatin (even though the blood culture for bacteria was negative) was used without success.

After 5 months on crizotinib treatment (October 2019), chest CT scan indicated that the size of primary tumor and lymph nodes at the hilum of left lung, intensity of obstructive pneumonia of left lung, and pleural effusion were progressed compared to that of August 2019. In addition, compared to the CT scan of August 2019, not only size of the cystic masses in the liver and kidneys was enlarged, but also the cystic masses in the left kidney invaded into the left psoas major muscle (Fig. 2E-H). With the suspicion of an infection, catheterized drainage of the cystic masses on the liver and left kidney was performed, respectively. Pathological examination of the liver drainage revealed negative for malignancy and demonstrated necrotic tissues of the liver with abscess and inflammatory cells. Histochemistry of the liver tissue revealed cytokeratin antibodies AE1/AE3 (-), calretnin (-), CD68 (+), and Wilms’ tumor 1 (WT-1, -). Pathological examination of the kidney drainage revealed inflammatory necrosis and negative malignancy. Crizotinib-associated renal cysts (CARC) was confirmed, and crizotinib was stopped on October 15th, 2019.

**Fig. 3:**
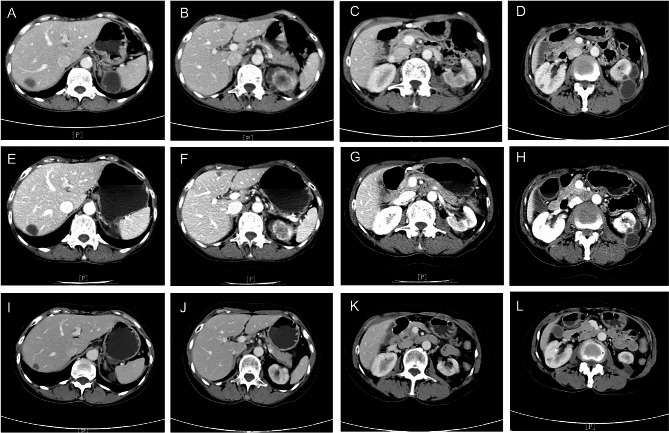
Serial contrast-enhanced abdominal CT scan after discontinuation of crizotinib. **Panels A-D**: One month after stopping crizotinib treatment, showing significant reduction of the size of cystic lesions. **Panels E-H**: Two months after stopping crizotinib treatment, showing continuous reduction of the cystic lesions. **Panels I-L**: Six months after stopping crizotinib treatment, demonstrating size of renal cysts significantly decreased and hepatic cyst nearly disappeared

**Fig. 4:**
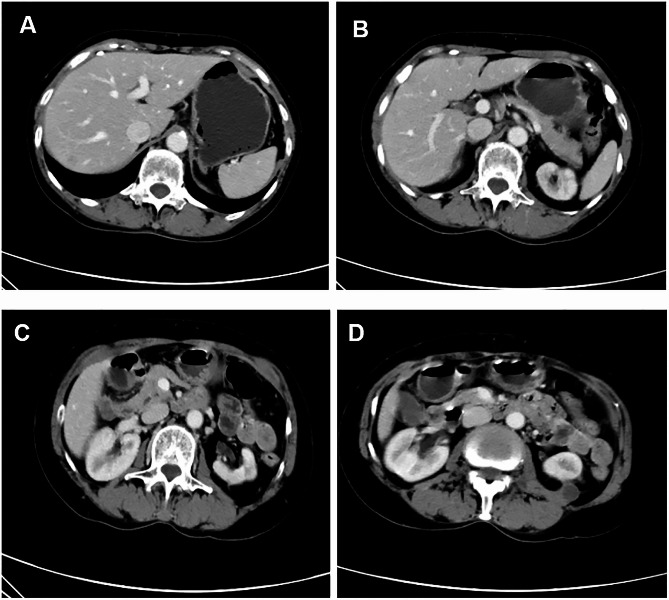
Contrast-enhanced abdominal CT scan after switching to alectinib. **Panels A-D**: Two months after switching the therapy to alectinib, showing no recurrence or enlargement of cystic lesions in the kidneys and liver

After one month of stopping crizotinib, the patient’s body temperature was back to normal, and the size of cystic masses in liver and kidneys significantly reduced, but hepatic subcapsular effusion was noted by CT scan (Fig. 3A-D). Patient was then followed up at 2 and 6 months after stopping crizotinib, and the size of cystic masses at kidneys and liver was continuously decreased (2 months: Figures 3E-H and 6 months: Fig. 3I-L) without noticeable change in the primary lung cancer at left hilum and metastatic tumors. Given the positive ALK fusion type, anti-cancer therapy was switched to alectinib (600 mg bid) 6 months after stopping crizotinib (April 31st, 2020). After 2 months of switching her treatment regimen to the 2nd generation ALK inhibitor, the regressed cystic lesions in the liver and kidneys remained unchanged (Fig. 4), and the size of primary lung cancer (approximately 2.6 × 1.9 cm) and lymph nodes on the left hilum as well as obstructive pneumonia were not changed, either.

## Discussion and conclusions

Crizotinib is the first generation of oral tyrosine kinase inhibitor that suppress the targets of ALK, ROS1, and c-met proto-oncogene product (c-MET, receptor of hepatic growth factor) [7–10, 15–17]. Introduction of crizotinib for the treatment of NSCLC with positive *ALK* rearrangement significantly extended progress-free survival (PFS) for the patients [18, 19]. However, development of crizotinib-associated renal cyst (CARC), a side effect generated by crizotinib through unknown molecular mechanisms, often prevents long-term application of crizotinib even though clear clinical benefits and partial or complete regression of NSCLC in radiographic imaging were achieved.

Although it has been reported that the incidence of CARC is low (4%) in general population [20], accumulating number of the case reports indicated that incidence of CARC seems higher in Asian compared to other Ethnic groups [21–24]. In this regard, as high as 23% (3 out of 13 patients) CARC has been reported in a report from Thailand [25], suggesting genetic background may contribute to the development of CARC.

The etiology and molecular mechanism of CARC formation remains unknown. While pathogenetic mechanism of autosomal dominant polycystic kidney disease (ADPKD) might be different from that of CARC, it has been reported that hepatocyte growth factor (HGF) and its receptor, c-MET, to be involved in the formation of ADPKD [26, 27]. In this regard, crizotinib is a dual c-MET and ALK inhibitor [10, 15–17], but accumulating number of case reports demonstrated that crizotinib is associated with development of cystic lesions in the kidneys, liver, and pancreas [20, 24, 28–35]. While the mechanism of CARC formation remains to be defined, as proposed by Wiest et al. [30], complete collection of the following information from the advanced NSCLC patients treated with crizotinib is recommended, that is, patient’s general information including age, gender, race/ethnicity, history of renal cysts; clinical information of primary including stage at diagnosis, genetic information, treatment history, treatment response, PFS; tyrosine kinase inhibitor-associated renal cysts including time to diagnosis, laterality, pathology, symptoms, complications, abnormalities in vitals and labs.

Natural history of CARC development is also unknown. Schnell et al. have summarized 17 published case reports of CARC and found that the “Time to diagnosis” ranged from 1.2 ∼ 15.2 months (Mean ± SD: 7.3 ± 4.3 months) [24]. In the current case report, cystic masses in both kidneys and liver were noted by the contrast-enhanced abdominal CT scan at 3 months after on crizotinib treatment when the patient was hospitalized due to low fever of unknown cause. Considering the possibility of metastatic tumors in the kidneys and liver as well as the clear result of regression of the primary lung cancer by radiographic imaging after initialization of crizotinib treatment, the patient in our case was continuously treated with crizotinib. However, we found that size of the cystic lesions in kidneys and liver continued to grow during the additional crizotinib treatment (from 3 months through 5 months on crizotinib). More importantly, biopsies of the kidney and liver turned out malignancy negative and confirmed diagnosis of CARC, which lead to discontinuation of crizotinib and switch to alectinib. Our experience suggested that, for the patients on crizotinib treatment, frequent radiographic inspection and prompt biopsy of the cystic mass in kidney and/or liver is strongly recommended to confirm diagnosis of CARC as soon as possible.

Misdiagnosis of CARC as metastatic renal malignancy or renal abscess could be encountered in clinic. In a retrospective analysis on incidence and patterns of evolution in 26 cases of CARCs, Cameron et al. [34] found that 4 out of 26 (15.4%) cases were falsely considered as malignancy or abscess. CARCs could be simple or complex cysts. A simple cyst can be asymptomatic, but complex CARCs can be complicated with infection, rupture, or abscess formation [36–38]. By abdominal CT scan, simple cysts are near water density with an upper threshold of 20 Hounsfield units (HU) [39], while complexing cysts could be hyperdense contents, septations, mixed solid cystic appearance, and poorly defined margins [38]. Complex CARCs fall into categories III and IV of Bosniak classification [40], which usually need surgical resection by standard guideline due to higher malignant potentiality. In this case, the imaging features are indistinguishable for differential diagnosis, and thus, histopathologic confirmation is necessary to exclude renal cell carcinoma or renal metastasis. In the current case report, metastatic renal malignancy was also considered in addition to renal cyst, and metastatic malignancies in kidney and liver were excluded through pathological examination showing malignancy negative in both renal and hepatic biopsy tissues, which showed granulation tissues with infiltration of inflammatory cells. Therefore, in addition to the serial radiographic imaging, pathological confirmation via tissue biopsy is recommended to avoid inappropriate treatment.

Majority of CARCs are spontaneously regressed dramatically or completely after discontinuation of crizotinib or switching to 2nd generation of ALK inhibitor, alectinib [32, 33]. Consistently, in the current case report, CARC was dramatically regressed two months after stopping crizotinib, and furthermore, cystic lesions in both kidneys and liver did not re-occur or progressed after switching to alectinib. Interestingly, Marino et al. have recently reported that alectinib could induce rapid regression of renal and hepatic cysts caused by crizotinib [32]. Similarly, Taima et al. reported that, 17 months after switching crizotinib to alectinib, not only CARC was significantly regressed, but also the hypoproteinemia and systemic inflammation were significantly improved [33]. The mechanism of these phenomenon, however, was not defined.

Taken together, while the development of genetic analysis of lung cancer and its targeting by an oral tyrosine kinase inhibitor, crizotinib, have revolutionized the treatment of NSCLC with positive *ALK* rearrangement, accumulating number of case reports indicate that crizotinib-associated renal cyst (CARC) leads discontinuation of crizotinib treatment even though it significantly prolongs progress-free survival (PFS) in the NSCLC patients. CARCs has features of malignancy and abscess in radiographic imaging, and thus, pathological confirmation through biopsy is necessary to avoid misdiagnosis and inappropriate treatment decision. In addition, it is of importance to define the molecular mechanism of CARC development in the future studies.

### Electronic supplementary material

Below is the link to the electronic supplementary material.

Supplementary Material 1

## Data Availability

The datasets used and/or analyzed during the current study are available from the corresponding author on reasonable request.

## Abbreviations

ALK: Anaplastic lymphoma kinase
CARC: Crizotinib-associated renal cyst
c-MET: c-MET proto-oncogene product
HGF: Hepatocyte growth factor
NSCLC: Non-small cell lung cancer
PFS: Progress-free survival
TKI: Tyrosine kinase inhibitor
